# A String of Pearls: Linear Immunoglobulin A (IgA) Bullous Dermatosis in the Setting of Imipramine Use and Newly Diagnosed Ulcerative Colitis

**DOI:** 10.7759/cureus.33448

**Published:** 2023-01-06

**Authors:** Samantha Davis, Mohamed Altattan, Aya Abugharbyeh, Yasmin Khader, Nezam Altorok

**Affiliations:** 1 Department of Internal Medicine, The University of Toledo, Toledo, USA; 2 Department of Rheumatology, The University of Toledo, Toledo, USA; 3 Internal Medicine, The University of Toledo, Toledo, USA

**Keywords:** dapsone treatment, corticosteroid treatment, ulcerative colitis (uc), drug -induced, autoimmune bullous dermatosis, string-of-pearls sign, vesiculobullous skin lesions, linear iga bullous dermatosis

## Abstract

Linear immunoglobulin A (IgA) bullous dermatosis (LABD) is an autoimmune disease affecting children or adults that leads to subepithelial vesiculobullous lesions on the skin and/or mucosa. Due to the histologic and clinical appearance of the disease with tense and pruritic blisters, direct immunofluorescence is required for diagnosis, which features the characteristic linear deposition of IgA autoantibodies along the basement membrane zone. LABD can be idiopathic, drug-induced, or associated with a systemic disease such as inflammatory bowel disease. Many drugs have been implicated, such as antibiotics, anti-hypertensives, anti-epileptics, analgesics, and immunosuppressive medications. Treatment of LABD centers on discontinuation of the offending drug, if applicable, as well as pharmacotherapy with dapsone as the first-line treatment. Adjunctive therapy with sulphonamides, systemic corticosteroids, cyclosporine, colchicine, intravenous immunoglobulins, tetracyclines, erythromycin, and dicloxacillin has also shown benefits. We report the case of a young adult patient who developed LABD with a background of recent initiation of treatment with imipramine and newly diagnosed ulcerative colitis.

## Introduction

Linear immunoglobin A (IgA) bullous dermatosis (LABD) is a rare autoimmune disease featuring subepithelial vesiculobullous lesions on the skin and/or mucous membranes [1]. LABD is characterized by IgA autoantibodies linearly deposited on the basement membrane zone with disruption of the dermo-epidermal junction leading to tense blisters; this is best visualized by direct immunofluorescence (DIF) and required for confirmatory diagnosis as the histologic and clinical appearance of the disease can resemble other conditions such as dermatitis herpetiformis [1-5]. Both children and adults can be affected. LABD is also known as a chronic bullous disease of childhood when it occurs in children with an average age of onset of 4.5 years old; for adults, the average age of onset typically occurs at one of two peaks: teenage to early adulthood or in the sixth decade [2,4,6]. The distribution of lesions is characteristically based on age, except for mucosal lesions, which can affect any age group. Children are more prone to developing a "string of pearls" sign with annular or polycyclic plaques and papules covering the ankles, thighs, buttocks, genitals, lower abdomen, wrists, and around the mouth or eyes. Adults more commonly develop lesions on the extremities, trunk, and head [1-2,4-6]. These lesions can cause varying degrees of pruritis [4-6]. Cases of LABD are typically idiopathic, drug-induced, or associated with a systemic disease such as ulcerative colitis, Crohn’s disease, systemic lupus erythematosus, rheumatoid arthritis, psoriasis, lymphoproliferative disorders, and infections [2,4,6]. We report a young adult patient with newly diagnosed ulcerative colitis and recent imipramine use who developed biopsy-proven LABD.

## Case presentation

An 18-year-old male with a history of epilepsy on levetiracetam, chronic constipation and abdominal pain since infancy, and chronic nocturnal enuresis since infancy started on imipramine one week prior and presented with a painful, itchy, diffuse skin eruption that worsened over 24 hours. The patient initially had tense vesicles on his neck, ears, and upper chest the previous day when evaluated by outpatient Allergy and Immunology. He was advised to discontinue imipramine due to concern for drug allergies and was prescribed empiric valacyclovir for herpes simplex virus and trimethoprim-sulfamethoxazole for bullous impetigo. However, the eruption then quickly spread to include his lower lip, abdomen, arms, and legs. The patient noted that the vesicles were tense and painful, especially after bursting. Additionally, the patient noted fatigue, generalized weakness, chronic constipation, abdominal pain in the bilateral lower quadrants with bowel movements, and occasional bright, blood-coated stools. The patient denied fever, chills, intraoral or esophageal lesions, odynophagia, dysphagia, arthralgia, myalgia, melena, nausea, vomiting, headache, and new exposures prior to the rash eruption, including poison ivy, new hygiene products, or new detergents.

**Figure 1:**
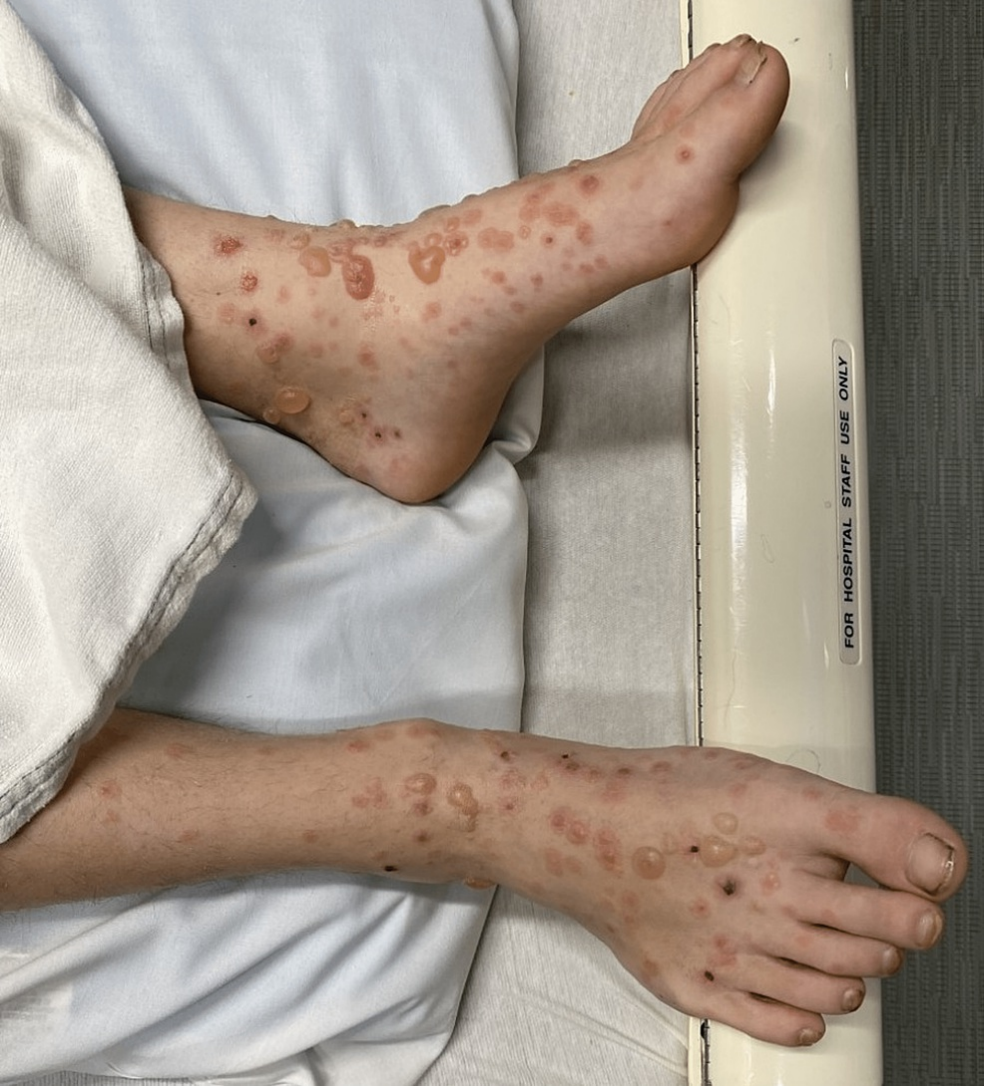
Lesions on the bilateral dorsal feet Bullous skin lesions in various stages of healing are seen on bilateral dorsal feet, sparing the soles.

**Figure 2:**
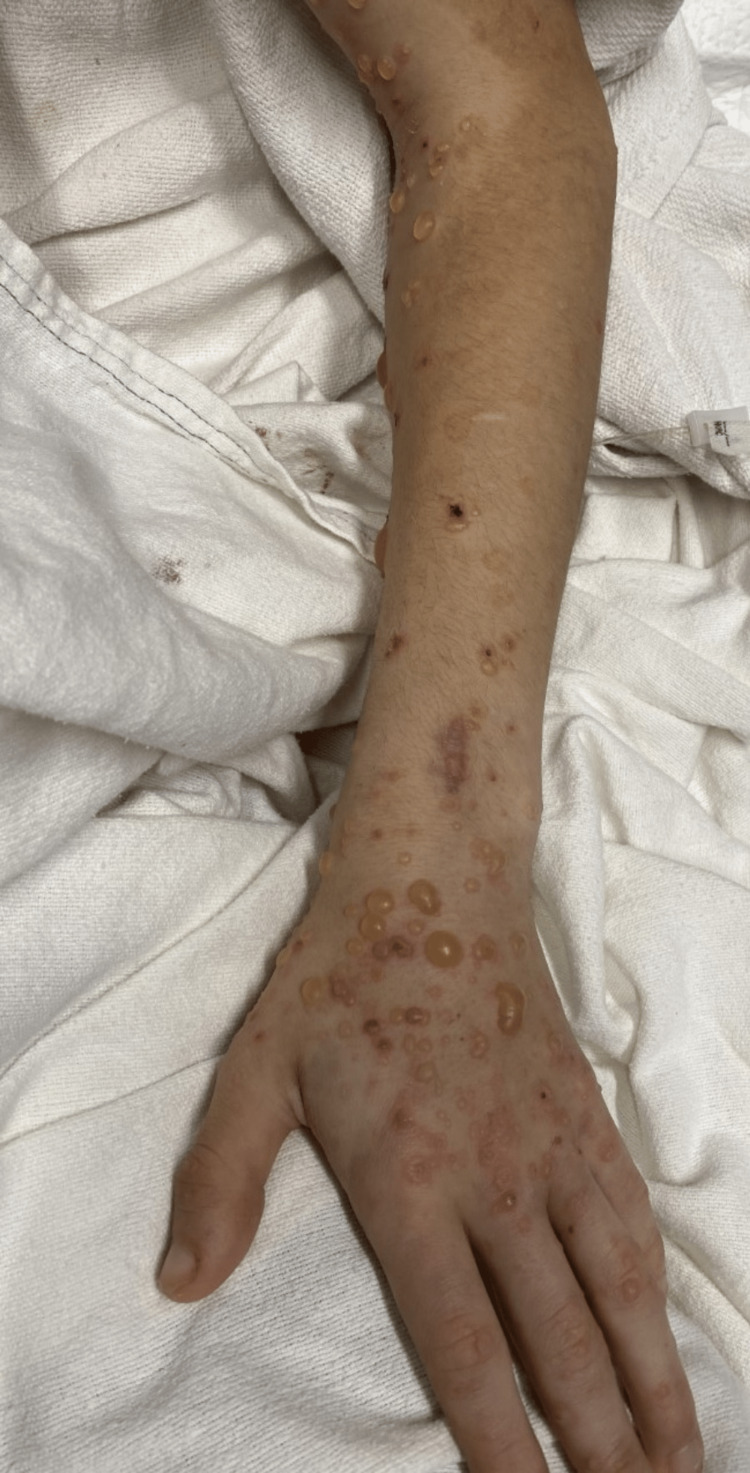
Lesions on the left upper extremity and dorsal hand Vesicular and bullous skin lesions in various stages of healing are demonstrated on the left upper extremity and dorsal hand, sparing the palm.

**Figure 3:**
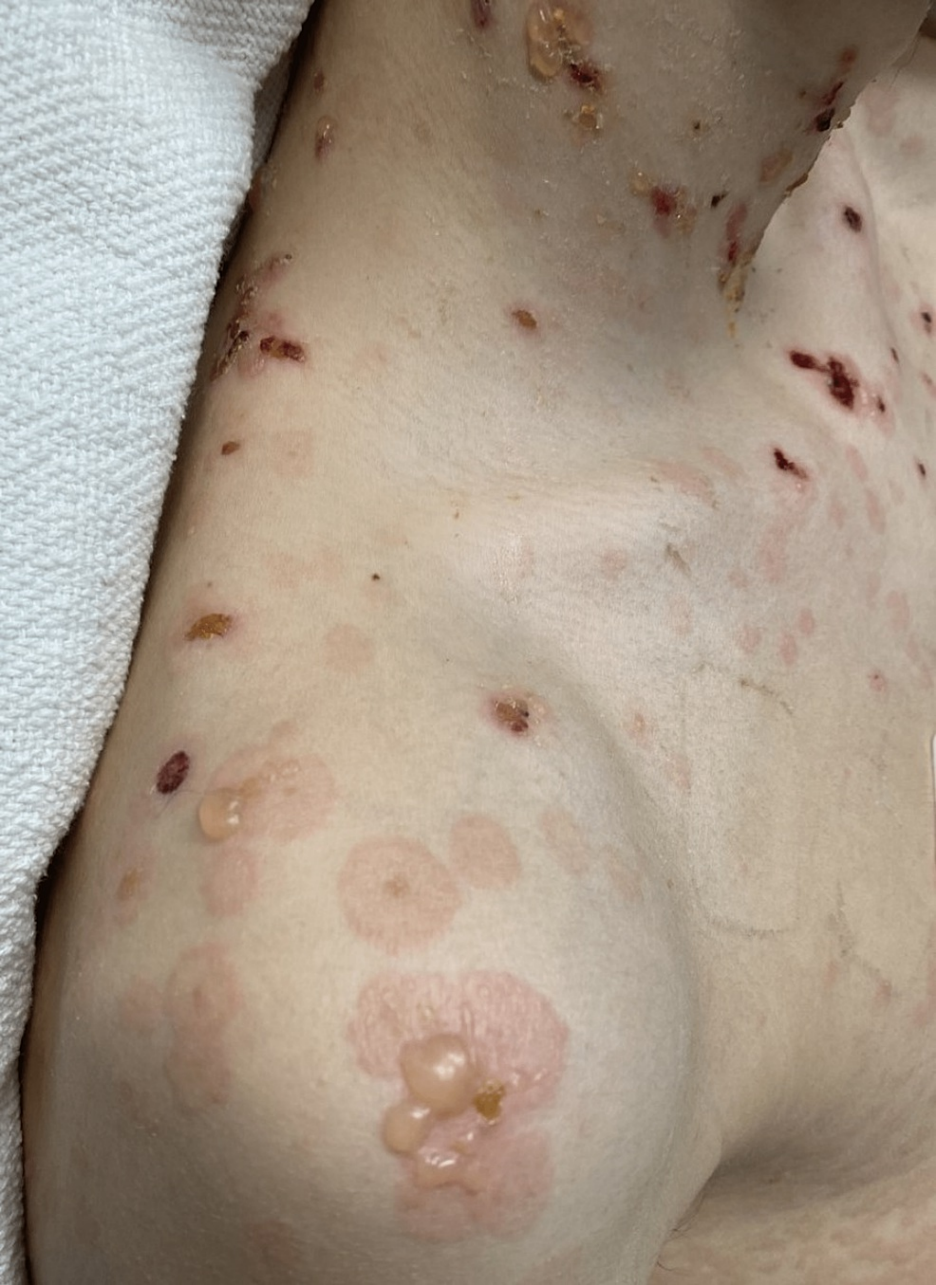
Lesions on the right neck, shoulder, and chest Bullous skin lesions on the right shoulder, right neck, and chest in various stages of healing.

**Figure 4:**
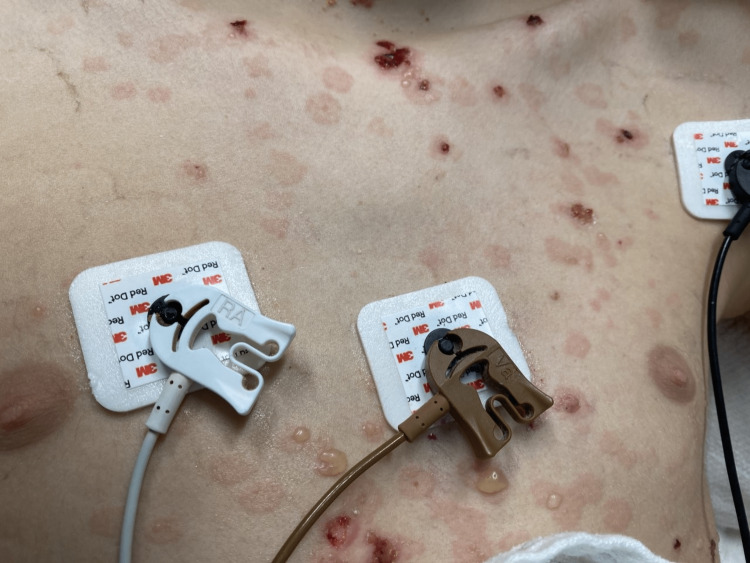
Lesions on the chest Healing and active bullous skin lesions are seen on the chest.

**Figure 5:**
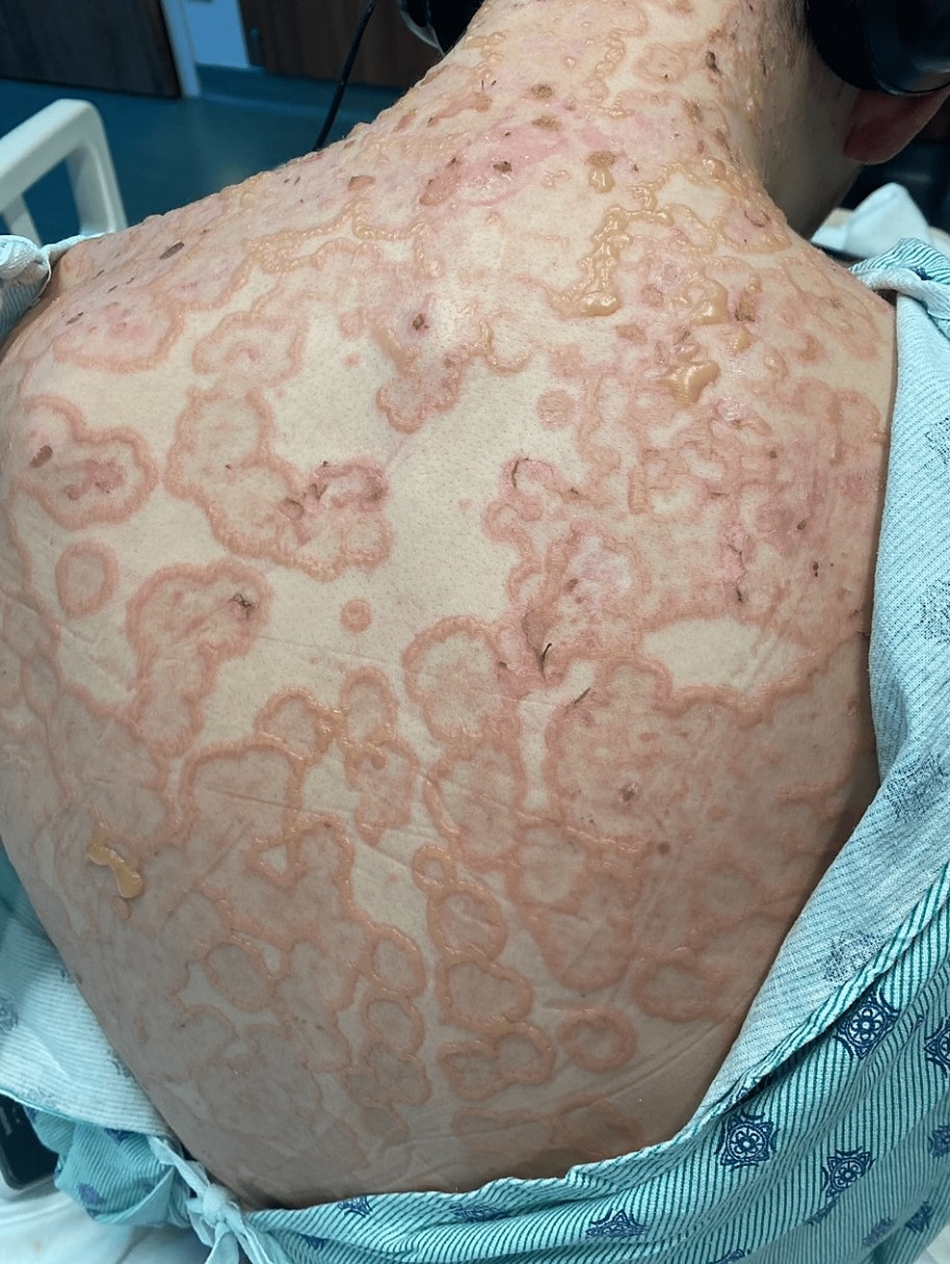
Lesions on the back and posterior neck with a “string of pearls” sign Active and healing bullous lesions on the back and neck coalesce to form annular rings known as the "string of pearls" sign.

**Figure 6:**
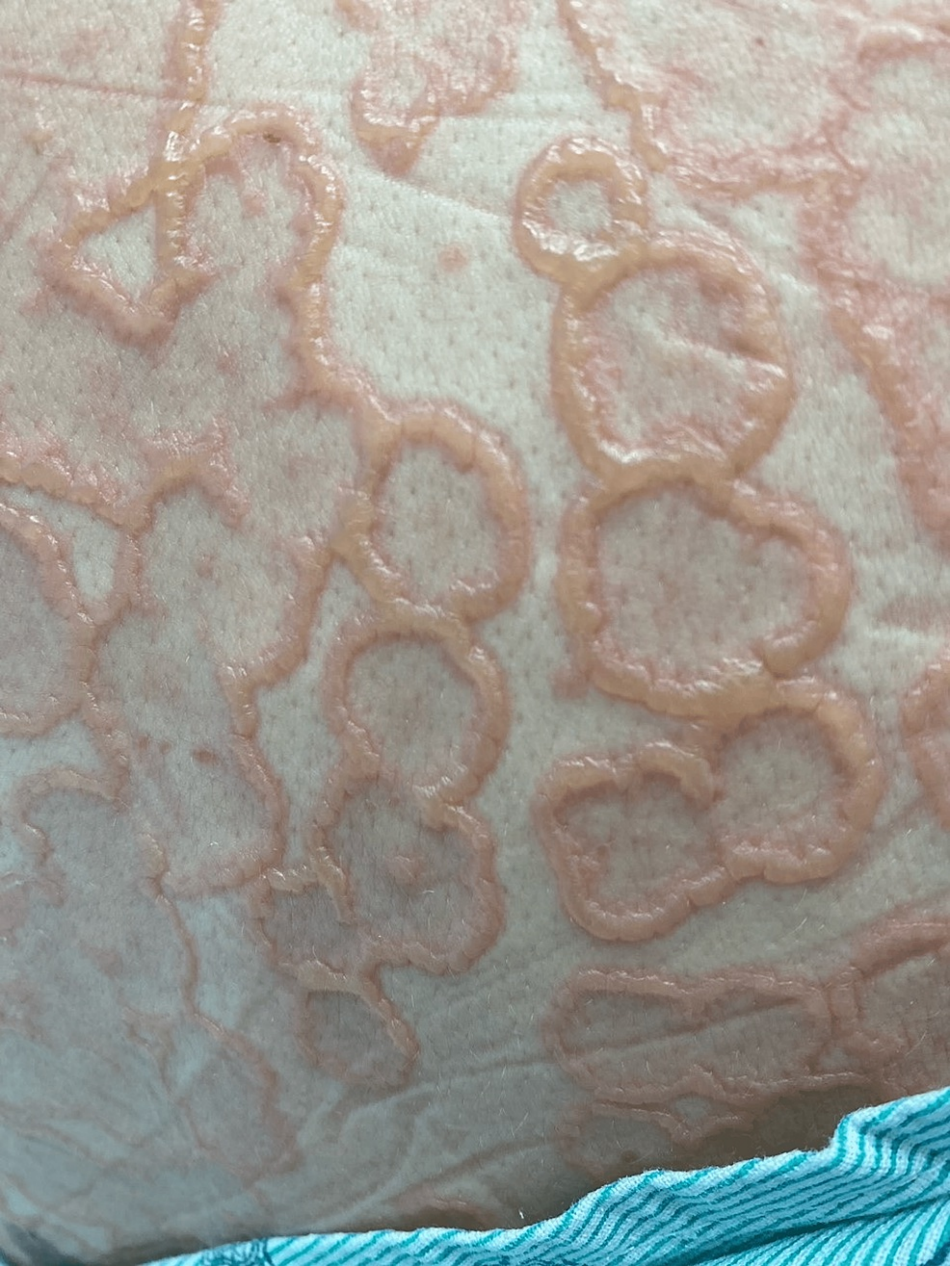
Close-up of the “string of pearls” sign from lesions on the back

Initial physical examination revealed the patient was afebrile, hemodynamically stable, with vesicles noted in the external auditory canals, moist mucous membranes without mucosal lesions, and a diffuse eruption with vesicles and bullae noted on the outer aspect of the lower lip, posterior neck, upper back, chest, arms sparing the palms, and legs sparing the soles. Some of the lesions appeared to be tense bullae, while others were less tense and umbilicated primarily on the dorsal hands and feet. Some of the lesions had burst, but most notably, there was a negative Nikolsky sign. Over the next few days, bullous lesions clustered in the classical pattern of a "string of pearls," as demonstrated in Figures 1-6.

Initial work-up included negative swabs via PCR for herpes simplex virus 1, herpes simplex virus 2, and varicella zoster, as well as a superficial wound culture of the posterior neck with rare Staphylococcus aureus. Laboratory findings included leukocytosis with eosinophilia, neutrophilia, mild monocytosis, microcytic anemia with iron deficiency, reticulocytosis, thrombocytosis, elevated inflammatory markers, elevated haptoglobin, and low ferritin (Table 1). The complete metabolic panel and lactate dehydrogenase levels were within normal limits (Table 1). The autoimmune workup was negative for the anti-nuclear antibody screening test, anti-double-stranded DNA antibodies, anti-Smith antibodies, anti-nuclear ribonucleoprotein antibodies, anti-Jo-1 antibodies (histidyl-tRNA synthetase), anti-Sjögren's-syndrome-related antigen A autoantibodies (anti-SSA/ro antibodies), anti-Sjögren's-syndrome-related antigen B (anti-SSB) autoantibodies, and scleroderma (Scl-70) antibodies. Complement component 3 (C3) and complement component 4 (C4) were within normal limits (Table 1). Immunoglobulin serum levels revealed elevated IgG1 subclasses (Table 1). Further work-up revealed negative results for the direct Coombs test, cold agglutinins, serum porphyrins, urine porphyrins, urine porphobilinogen, and 6-methylmercaptopurine. The glucose-6-phosphate dehydrogenase level was within normal limits (Table 1). A peripheral smear conducted during the hospitalization revealed mild neutrophilia attributed to the treatment plan, microcytic anemia secondary to iron deficiency and blood loss, reticulocytosis for bone marrow compensation, and reactive thrombocytosis attributed to anemia. Flow cytometry of peripheral blood had no abnormal findings. The infectious work-up was negative for HIV, hepatitis B, hepatitis C, and tuberculosis via QuantiFERON. A gastroenterological work-up revealed a positive fecal occult blood test (FOBT), significantly elevated fecal calprotectin, and negative transglutaminase IgA antibodies (Table 1). The patient had mild zinc insufficiency and an elevated vitamin B12 level, but levels for vitamin E, chromium, niacin, vitamin B6, folate, and copper were within normal limits (Table 1). Two skin-punch biopsies of the right posterior shoulder revealed a subepidermal cleft or blister with neutrophils at the tips of the dermal papillae. Direct immunofluorescence testing of the skin biopsies revealed continuous, strong linear basement membrane deposition of IgA, consistent with linear IgA bullous dermatosis.

**Table 1:** Laboratory values

Laboratory Study	Value	Reference Range
White Blood Cells (WBCs)	14.2 x 10^9/L	4.0-11.0 x 10^9/L
Neutrophils, absolute	7.9 x 10^9/L	1.5-6.6 x 10^9/L
Lymphocytes, absolute	3.5 x 10^9/L	1.0-3.5 x 10^9/L
Monocytes, absolute	1.3 x 10^9/L	0.0-0.9 x 10^9/L
Eosinophils, absolute	1.3 x 10^9/L	0.0 - 0.4 x 10^9/L
Basophils, absolute	0.1 x 10^9/L	0.0-0.2 x 10^9/L
Hemoglobin	9.7 g/dL	13.0-17.0 g/dL
Mean corpuscular volume (MCV)	77 fL	80-100 fL
Iron	21 ug/dL	50-212 ug/dL
Total iron binding capacity (TIBC)	465 ug/dL	250-425 ug/dL
Iron saturation	5%	20-50%
Ferritin	7 ng/mL	24-336 ng/mL
Reticulocytes	3%	0.4-2.2 %
Platelets	532 x 10^9/L	150-450 x 10^9/L
C-reactive protein	1.8 mg/dL	0-0.744 mg/dL
Erythrocyte sedimentation rate	20 mm/h	0-15 mm/h
Haptoglobin	257 mg/dL	32-228 mg/dL
Lactate dehydrogenase	199 U/L	100-235 U/L
C3 complement	161 mg/dL	86-184 mg/dL
C4 complement	31 mg/dL	16-47 mg/dL
Serum Immunoglobulin A (IgA)	229 mg/dL	68-378 mg/dL
Serum Immunoglobulin M (IgM)	106 mg/dL	45-281 mg/dL
Serum Immunoglobulin G (IgG)	1,597 mg/dL	635- 1,741 mg/dL
Serum Immunoglobulin G1 (IgG1)	1,175.8 mg/dL	315 - 855 mg/dL
Serum Immunoglobulin G2 (IgG2)	199.1 mg/dL	64-495 mg/dL
Serum Immunoglobulin G3 (IgG3)	157.4 mg/dL	23-196 mg/dL
Serum Immunoglobulin G4 (IgG4)	33.9 mg/dL	11-157 mg/dL
Cold agglutinins	<1:32 dilutions	<1:32 dilutions
Serum porphyrins	<10 nmol/L (negative)	0-15 nmol/L
Urine porphyrin – Uroporphyrin	1 umol/mol	0-4 umol/mol
Urine porphyrin – Heptacarboxylate	0 umol/mol	0-2 umol/mol
Urine porphyrin – Coproporphyrin I	2 umol/mol	0-6 umol/mol
Urine porphyrin – Coproporphyrin III	7 umol/mol	0-14 umol/mol
Urine porphobilinogen, quantitative	<5 umol/L	0.0-8.8 umol/L
6-methylmercaptopurine	<310 pmol/8 x 10^8 RBC	< or = 5700 pmol/8 x 10^8 RBC
Glucose 6 phosphate dehydrogenase, quantitative	13.9 U/g Hb	9.8-15.5 U/g Hb
Sodium	137 mmol/L	134-146 mmol/L
Potassium	4.2 mmol/L	3.5-5.0 mmol/L
Chloride	107 mmol/L	98-109 mmol/L
Bicarbonate	26 mmol/L	22-36 mmol/L
Blood Urea Nitrogen (BUN)	11 mmol/L	5-23 mmol/L
Creatinine	0.82 mg/dL	0.30-1.00 mg/dL
Glomerular Filtration Rate (GFR)	>60 mL/min/1.73 sq.m	>59 mL/min/1.73 sq.m
Glucose	101 mg/dL	65-99 mg/dL
Calcium	8.3 mg/dL	8.5-10.5 mg/dL
Total Protein	6.5 g/dL	6.0-8.0 g/dL
Albumin	3.3 g/dL	3.2-5.3 g/dL
Alkaline Phosphatase	110 U/L	39-130 U/L
Aspartate Transaminase (AST)	22 U/L	0-41 U/L
Alanine Transaminase (ALT)	29 U/L	0-40 U/L
Total Bilirubin	0.4 mg/dL	0.3-1.2 mg/dL
Zinc	54 ug/dL	60-120 ug/dL
Vitamin B12	1,455 pg/mL	180-914 pg/mL
Folate	6.8 ng/mL	>5.8 ng/mL
Vitamin E, Alpha-Tocopherol	6.1 mg/L	5.5-18.0 mg/L
Vitamin E, Gamma-Tocopherol	1.0 mg/L	0.0-6.0 mg/L
Chromium	1.2 ug/L	<5.0 ug/L
Niacin	1.36 ug/mL	0.50-8.45 ug/mL
Vitamin B6	21.1 nmol/L	20.0-125.0 nmol/L
Copper	129 ug/dL	70-140 ug/dL
Fecal calprotectin	>3,000 mg/kg	>120 mg/kg

**Figure 7:**
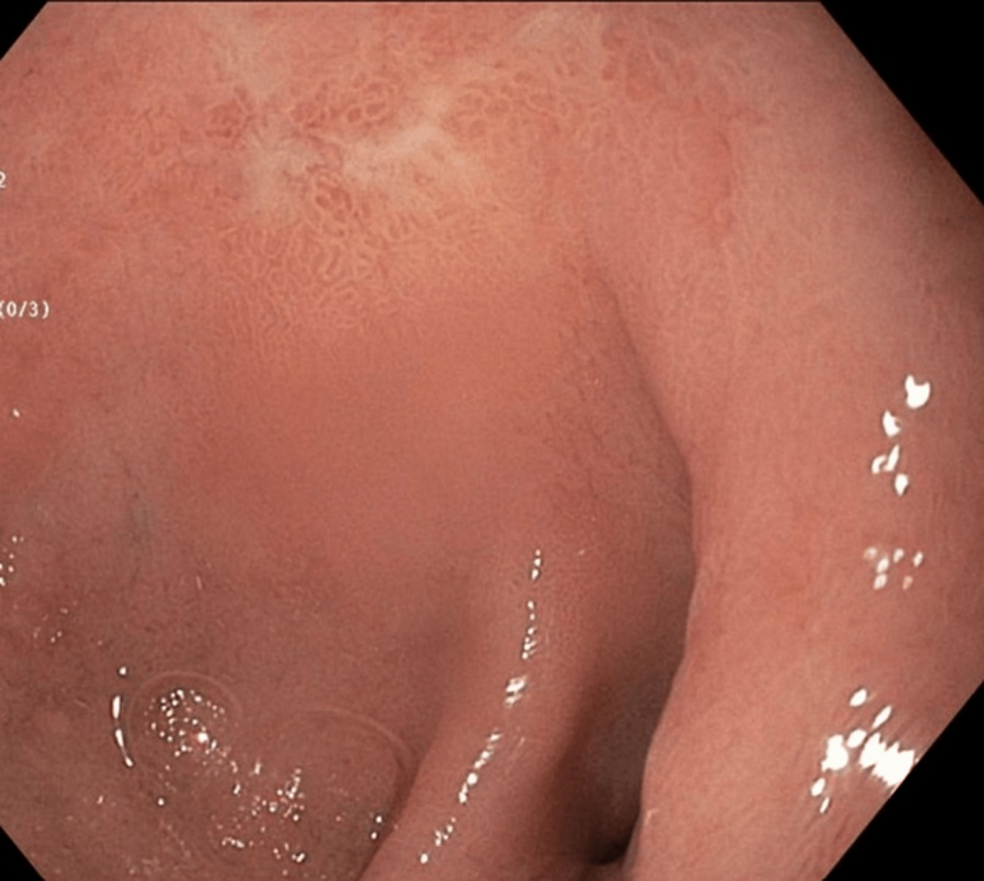
Duodenal bulb erosion found on esophagogastroduodenoscopy (EGD) On EGD, erosion without any signs of bleeding was found in the duodenal bulb, as depicted above and best seen in the upper third of the figure.

**Figure 8:**
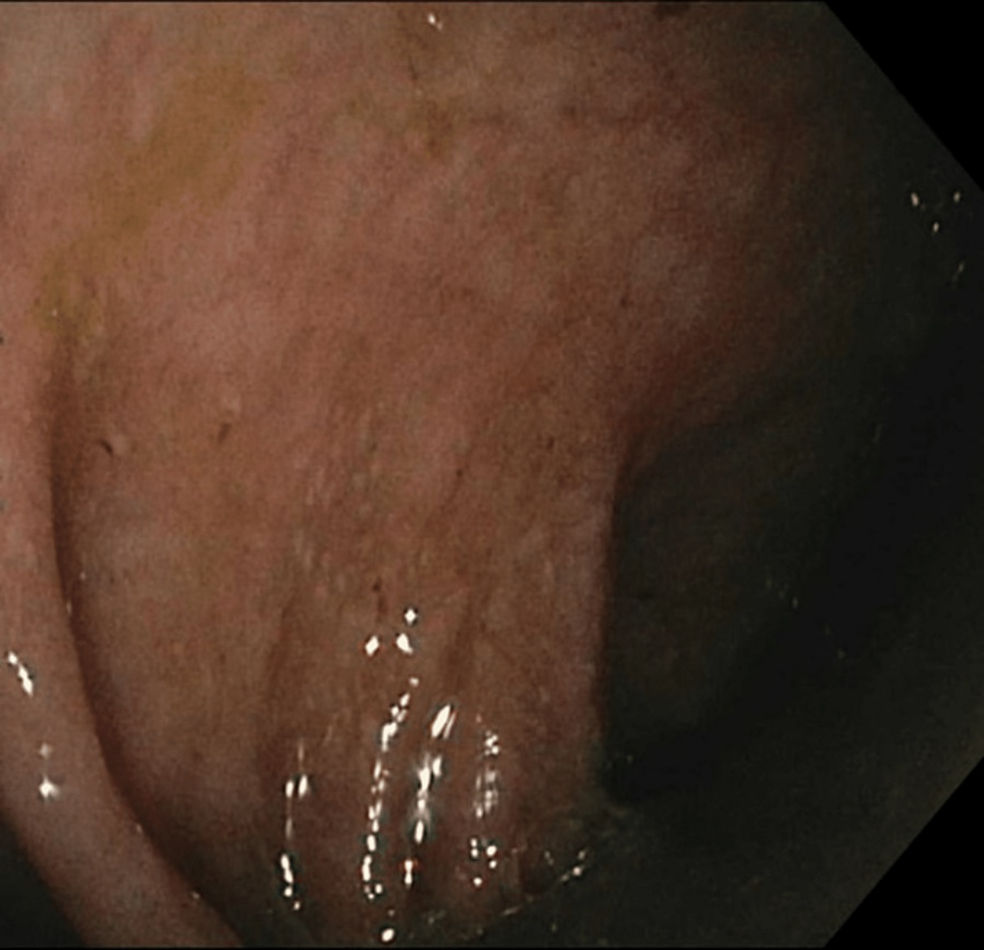
Active ulcerative colitis in the descending colon was found on colonoscopy The colonoscopy revealed a Mayo Score of 2 and active ulcerative colitis in the descending colon.

**Figure 9:**
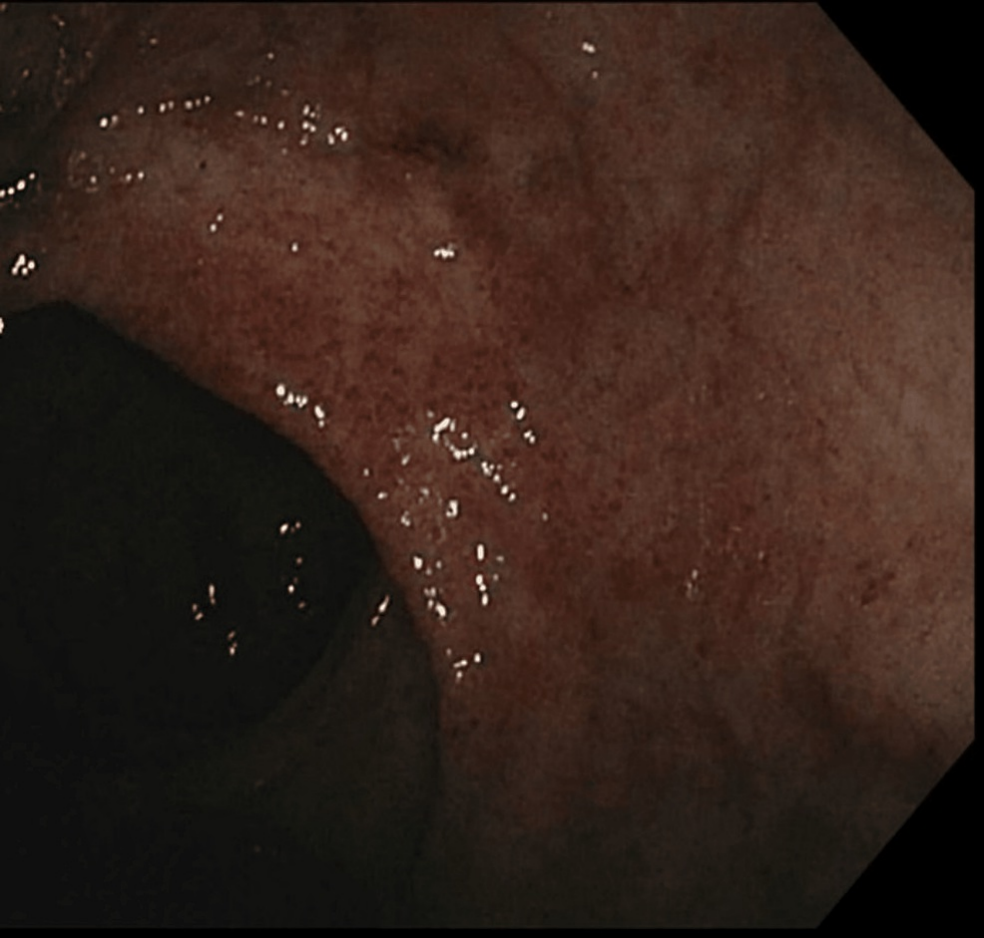
Active ulcerative colitis in the rectum was found on colonoscopy The colonoscopy revealed a Mayo Score of 2 and active ulcerative colitis in the rectum. Erythema can be seen in the center of the figure.

The patient underwent esophagogastroduodenoscopy (EGD) and colonoscopy; EGD revealed a non-bleeding duodenal erosion, and duodenal biopsies were taken (Figure 7). Duodenal biopsies revealed duodenal bulb mucosa with gastric foveolar metaplasia consistent with mild peptic injury and notably negative for signs of celiac disease, malignancy, viral inclusions, and granuloma. Colonoscopy revealed moderately active (Mayo Score 2), left-sided ulcerative colitis with random colonic biopsies taken (Figures 8, 9). Random biopsies of the left colon demonstrated chronic, moderately active colitis, while random right colon biopsies revealed focal, chronic, but inactive colitis.

Imipramine was discontinued before hospitalization and not resumed due to the suggestion of drug-induced dermatosis. The patient was started on oral prednisone 10 mg when eruption failed to improve with supportive care after receiving one dose. The corticosteroid regimen was up-titrated the following day for worsening pain to methylprednisolone 125 mg intravenously twice daily, for which the patient received four days of treatment. As the eruption had improved, the patient was weaned to oral prednisone 40 mg daily, receiving two days of therapy before the eruption worsened again. Oral prednisone was increased to 60 mg with the addition of oral azathioprine 50 mg twice daily. Despite this, the eruption worsened, which necessitated further up-titration of corticosteroids to methylprednisolone 500 mg intravenously daily for three days while continuing azathioprine at the current dose. Treatment with oral dapsone was initiated, partly as a steroid-sparing medication for LABD and partly for Pneumocystis jirovecii prophylaxis. After completion of the intravenous methylprednisolone three-day course, the patient was transitioned to oral methylprednisolone 64 mg daily, at which point azathioprine was also stopped. Treatment with infliximab was initiated after discharge due to findings of ulcerative colitis on a colonoscopy. The patient was ultimately discharged on oral methylprednisolone, dapsone, and infliximab infusion as an outpatient. On follow-up four weeks and three months later, the patient had complete resolution of skin lesions, with moderate pigmentation at the site of some previous skin lesions but no scars and no abdominal pain.

## Discussion

We report a case of newly diagnosed ulcerative colitis (UC) with recent imipramine use for chronic nocturnal enuresis who subsequently developed biopsy-proven linear IgA bullous dermatosis and was found to also have UC. The patient was successfully treated with a corticosteroid regimen and dapsone during his inpatient stay, followed by infliximab.

The pathogenesis of linear IgA bullous dermatosis involves circulating IgA autoantibodies targeting the basement membrane zone specifically at the lamina lucida’s BP180, BP280, and LAD285 molecules. For BP180, also known as bullous pemphigoid antigen 2 (BPAG2), autoantibodies tend to target the 97 kDa subunit at the NC16A domain or the 120 kDa/97 kDa ectodomain of the molecule in immunobullous diseases such as bullous pemphigoid or LABD. Collagen type 7 has also been identified as an IgA autoantibody target, particularly in the sub-lamina densa variant of LABD, though the complete pathophysiology is not yet well understood [4,6]. The pathogenesis of drug-induced LABD has pointed to culprit drugs inducing an autoimmune response via cross-reactivity with epitopes, change of epitope conformation, or exposure of formerly sequestered antigens. However, there is also a suggestion of T-cell and cytokine involvement. Cytokines from drug-specific T-cells, such as interleukins IL-4, IL-5, IL-6, IL-10, and transforming growth factor β have been implicated in increasing IgA synthesis. Cytotoxic CD8+ lymphocytes also have been presumed to incite the initial autoantigen recognition for drug-induced LABD [4].

Linear IgA bullous dermatosis can be idiopathic, drug-induced, or associated with systemic disease. As mentioned above, there are many systemic diseases that have been associated with LABD: rheumatoid arthritis, psoriasis, systemic lupus erythematosus, dermatomyositis, multiple sclerosis, celiac disease, Crohn’s disease, and UC [4,6]. Ulcerative colitis (UC) has a very frequent association with LABD [4]. UC manifestation often occurs before the onset of LABD in the majority of UC patients who encounter this blistering disease, due to the proposed mechanism of intestinal inflammation, which exposes and cross-reacts intestinal and cutaneous antigens to produce an autoimmune response at the basement membrane zone [7]. In addition, LABD has also been weakly associated with malignancy, especially lymphoproliferative disorders and cancer of the esophagus, thyroid, colon, and kidney [6]. Bladder cancer has been directly associated as well [4]. Lastly, idiopathic LABD has been linked to some infections, such as upper respiratory tract infections, gynecological infections, typhoid, tetanus, brucellosis, and varicella-zoster [4]. Though varicella-zoster virus infection can lead to chickenpox or shingles eruptions, it may play a role in the formation of IgA autoantibodies like other respiratory infections [6].

Drug-induced LABD represents a significant portion of cases for adult patients [2,4,6]. Typically, the disease manifests within one month of drug administration [6]. There are many classes of medications that have been implicated in causing LABD: antibiotics, anti-hypertensives, anti-epileptics, analgesics, and immunosuppressive medications. Regarding antibiotics, vancomycin is the most common drug to incite LABD in patients [2,4,6]; however, other antibiotics have been implicated, such as penicillins, cephalosporins, metronidazole, moxifloxacin, rifampicin, and sulfonamides, with especially trimethoprim-sulfamethoxazole as another common inciting drug. Antihypertensives implicated include angiotensin-converting enzyme inhibitors like captopril, angiotensin receptor blockers like candesartan, and calcium channel blockers like verapamil and amlodipine. Amiodarone, atorvastatin, furosemide, allopurinol, and glyburide have also been reported. Antiepileptics incriminated include vigabatrin and lithium carbonate, with phenytoin being the most common antiepileptic cited among them. Analgesics known to cause LABD include acetaminophen, buprenorphine, and non-steroidal anti-inflammatory drugs (NSAIDs) like diclofenac, naproxen, ketoprofen, and piroxicam [4,6]. Immunosuppressive, immunomodulatory, and antineoplastic medications have also been cited; these include gemcitabine, sulfasalazine, interferon-γ/interleukin-2, interferon-α 2a, cyclosporine, granulocyte colony-stimulating factor, and infliximab. Infliximab specifically has been paradoxically linked to outbreaks of LABD in inflammatory bowel disease patients [4]. Interestingly, influenza vaccination has also been reported as an inciting agent [6]. Despite the numerous drugs identified in triggering linear IgA bullous dermatosis, it has been suggested that drug administration and eruption onset need to be correlated since drug-induced LABD can occur in patients with underlying neoplastic disease, diabetes, heart disease, pulmonary disease, or systemic disease. This has been particularly reported with bladder cancer, rheumatoid arthritis, and UC [4].

Treatment of LABD largely centers on pharmacotherapy but also must consider instigating agents. Drug-induced LABD treatment starts with discontinuation of the offending drug, which can result in gradual, spontaneous resolution of the eruption within several weeks [2,4]. However, for up to half of patients, drug discontinuation must be paired with additional therapy to calm the immunological cascade [4]. Dapsone has emerged as the first-line therapy for LABD [1-2,4,6]. At comparatively low doses, dapsone effectively treats the lesions, with improvement commonly noted within 48-72 hours of administration. Dapsone is typically dosed at 1-2 mg/kg for children and 100 mg on average for adults [2,6]. Before and while on dapsone therapy, patients should be monitored with liver function tests and a complete blood cell count with differential [2]. Patients should also be closely monitored for leukopenia, agranulocytosis, hemolytic anemia, dapsone hypersensitivity syndrome, peripheral neuropathy, nephrotic syndrome, and cholestatic jaundice [6]. It is also important to check the patient for glucose-6-phosphate dehydrogenase deficiency due to the risk of severe hemolytic anemia with dapsone administration in those patients [2]. For cases that need adjunctive or alternative treatment, there has been reported use of sulphonamides like sulfapyridine, systemic corticosteroids, cyclosporine, colchicine, and intravenous immunoglobulins [1-2,4,6]. Nicotinamide has also been reported to be beneficial [2,6]. The addition of systemic therapy with gradual tapering toward treatment termination may be required until induction of remission [2]. Additionally, antibiotics such as erythromycin, tetracyclines, dicloxacillin, and trimethoprim-sulfamethoxazole have also shown benefit, though no identifiable underlying infectious etiology to be targeted has been elucidated [1-2,6]. For patients with specifically drug-induced LABD from vancomycin, doxycycline administration after discontinuation of vancomycin has interestingly shown some benefit [6]. Unfortunately, for patients with idiopathic LABD, it is possible for the disease to remit and relapse over a decade or longer despite treatment [2].

## Conclusions

In conclusion, we present a case of biopsy-proven linear IgA bullous dermatosis associated with newly diagnosed ulcerative colitis and imipramine use. Regarding this case, it is challenging to truly isolate the primary etiology of LABD in this patient. The etiology could be a purely ulcerative colitis-induced LABD that is triggered to manifest by imipramine due to the timing of the initiation of this medication and the onset of skin manifestations. LABD has a frequent association with ulcerative colitis; however, imipramine has yet to be identified as a causative drug in the literature. We suggest that this patient likely has drug-induced linear IgA bullous dermatosis from imipramine with underlying ulcerative colitis.
